# Trends in Unhealthy Lifestyle Factors among Adults with Stroke in the United States between 1999 and 2018

**DOI:** 10.3390/jcm12031223

**Published:** 2023-02-03

**Authors:** Yuting Liu, Haochen Wang, Bingqing Bai, Fengyao Liu, Yilin Chen, Yu Wang, Yanting Liang, Xiaohe Shi, Xueju Yu, Chao Wu, Lan Guo, Huan Ma, Qingshan Geng

**Affiliations:** Department of Cardiology, Guangdong Cardiovascular Institute, Guangdong Provincial People’s Hospital, Guangdong Academy of Medical Sciences, Southern Medical University, Guangzhou 518000, China

**Keywords:** secondary prevention, stroke, lifestyle, depression, smoking, exercise

## Abstract

**BACKGROUND:** Unhealthy lifestyle factors are risk factors for stroke, and they play a key role in stroke secondary prevention. A better understanding of these factors may aid with improvements in public health policy. **OBJECTIVE:** Our objective was to comprehensively understand the trends in unhealthy lifestyle factors in people who have previously had a stroke in the US. **METHODS:** Utilizing data from the biannual United States National Health and Nutrition Examination Surveys (NHANESs) between 1999 and 2018, we collated data on unhealthy lifestyle factors (smoking, alcohol drinking, depression, unhealthy diet, high BMI, physical inactivity, and sedentary behavior) in adults with a history of stroke. The Joinpoint Regression model was used to calculate the annual percentage change (APC) and average annual percentage change (AAPC) to identify trends. Logistic regression modeling was used to identify the influence of sociodemographic factors (age, sex, race/ethnicity, marital status, employment status, family income, and highest education level). **RESULTS:** The analysis included 2017 respondents with a history of stroke. Current alcohol drinking (39.3% (95% confidence interval: 29.8, 48.7) to 57.4% (45.7, 69.0) *p* = 0.008) and obesity (39.2% (28.3, 50.2) to 49.4% (38.9, 59.8) *p* = 0.029) increased significantly from 1999 to 2018. The prevalence of smoking and depression remained generally stable. The proportion of respondents with an unhealthy diet decreased from 1999 (44.5% (32.4, 56.5)) to 2011 (29.0% (17.5, 40.4) *p* = 0.019), but then returned to its original prevalence in 2018 (42.0% (31.4, 52.7)). From 2007 to 2018, the proportion of respondents who were physically inactive decreased significantly, from 70.4% (64.4, 76.3) to 55.1% (46.1, 64.2; *p* = 0.017). After a gradual increase in sedentary activity from 2007 to 2012, this declined from 2013 to 2018, with no statistical significance. We found stroke survivors who were widowed, divorced, separated, or unemployed were at a higher risk of having unhealthy lifestyles than those who were employed or had other marital statuses. **CONCLUSIONS:** A modest reduction in the prevalence of physical inactivity was observed in Americans with a history of stroke between 1999 and 2018. The prevalences of smoking, drinking, depression, poor diet, obesity, and sedentary behavior were stable or increasing.

## 1. Introduction

Stroke is associated with notable healthcare costs, loss of productivity, diminished quality of life, and mortality in the United States (US) [1]. Modifiable lifestyle factors play a key role in secondary stroke prevention [2]. Clear evidence shows that adhering to a healthy lifestyle and controlling the elements described above lowers the risk of stroke-related illness, recurrent strokes, and poor cardiovascular outcomes [3,4,5]. However, to date, there has been no comprehensive estimation of the temporal trends in unhealthy lifestyle factors among those with a history of stroke. This study aimed to use data from the nationally representative and biannual National Health and Nutrition Examination Surveys (NHANESs) from 1999 to 2018 to demonstrate the trends in lifestyle factors in people who have previously had a stroke and who reside in the US. Further, we identified population subgroups which were at a higher risk of unhealthy factors. The unhealthy lifestyle factors of this study were defined as smoking, alcohol drinking, depression, unhealthy diet, obesity, physical inactivity, and sedentary behavior, which were thought to be most associated with the occurrence, development, and prognosis of stroke or cardiac–cerebral disease according to previous studies and guidelines [5,6,7,8]. It would be of great interest to investigate the trends of unhealthy lifestyle factors among stroke survivors to inform current public health priorities and provide guidance for future disease prevention and long-term health promotion strategies, in both the U.S. and other countries.

## 2. Methods

### 2.1. Sample

The NHANES is a biannual survey series designed to assess the health and nutritional status of the U.S. population using a complex, stratified, probability cluster method to generate nationally representative statistics. The National Center for Health Statistics Research Ethics Review Board approved the study protocol and all respondents provided written informed consent. The research followed the Declaration of Helsinki.

This analysis used cross-sectional data for NHANES respondents from 1999 to 2018 with a history of stroke who were aged ≥20 years. Respondents were asked if they had a history of a stroke diagnosed by a physician or other health professional. Self-reporting of strokes is a validated method in the general population and has been used in many large-scale epidemiological studies of stroke survivors [9]. Respondents who did not participate in the physical examination were excluded.

### 2.2. Unhealthy Lifestyle Factors

We collated data for the unhealthy lifestyle factors of smoking, alcohol drinking, depression, unhealthy diet, high body mass index (BMI)/obesity, physical inactivity, and sedentary behavior. Information for depression was available from 2005–2006 through 2017–2018. Information for physical inactivity and sedentary behavior was available from 2007–2008 through 2017–2018.

We defined the unhealthy lifestyles of our study according to the definitions of the poor or intermediate level in the AHA 2020Goals [5] and other references, as follows.

Current smokers were defined as those who had smoked ≥100 cigarettes over their lifetime, currently smoked cigarettes, or had done so in the last 5 days [5]. Current alcohol drinkers were defined as those who had consumed ≥1 alcohol drink in the past 12 months (for all surveys) or ≥12 drinks in their lifetime (for all but 2017–2018 survey, due to a change in the questionnaire) [7]. Depression was defined by a Patient Health Questionnaire-9 (PHQ-9) score of ≥10 [10]. 

AHA diet secondary scores were calculated according to 5 primary dietary components and 3 secondary dietary components, namely total consumption of fruits and vegetables; fish and shellfish; sodium; sugar-sweetened beverages; whole grains, nuts, seeds, and legumes; processed meat; and saturated fat. An unhealthy diet was defined by an AHA diet secondary score <32 (<40% of optimal score). All dietary variables were energy-adjusted to 2000 kcal/day using a residual method prior to analysis [11]. Obesity was defined as a BMI ≥30 kg/m^2^. Physical inactivity was defined as <150 min/week of moderate or <75 min/week of vigorous activity, or <150 min/week of moderate and vigorous activity [5]. Sedentary behavior was consisted of ≥6 h/day of sedentary behavior during waking hours [12].

The number of lifestyle factors considered ‘unhealthy’ was summed for each respondent from 2007–2008. Prior to this, data were not available for all factors. These were then given an overall lifestyle health score: 0 = ideal, 1–2 = good, 3–4 = intermediate, 5–6 = poor, or 7 = extremely poor.

### 2.3. Sociodemographic Variables 

The effect of several sociodemographic variables on the risk of unhealthy lifestyle factors was assessed. These variables included self-reported age (grouped as 20–44, 45–64, or ≥65 years), sex (male or female), race/ethnicity (grouped as non-Hispanic White, non-Hispanic Black, Hispanic, or other), family income (calculated and grouped as a poverty to income ratio (PIR) <1.30, 1.30 to 3.49, or ≥3.50), education level (grouped as below high school, high school graduate/General Equivalency Diploma, or some college/above), marital status (grouped as married/living with partner, never married, or widowed/divorced/separated), and employment status (unemployment or employment).

### 2.4. Analyses

The age-adjusted proportion of respondents and a 95% confidence interval (CI) were determined for each of the demographic, socioeconomic, and lifestyle-related characteristics using data from each of the ten NHANES from 1999–2000 to 2017–2018. We obtained standard errors and CI using the Taylor series (linearization) method, following analytic procedures. To identify significant changes in unhealthy lifestyle trends over the course of the surveys (1999 to 2018), we used a Joinpoint Regression model to calculate the annual percentage change (APC) and average annual percentage change (AAPC) over the entire period. All NHANES survey data were treated as a single combined cross-section when evaluating the relationships between sociodemographic variables and unhealthy lifestyle factors. Logistic and multinomial logistic regression models were used to obtain odds ratios (ORs). The odds ratios for depression, physical inactivity, and sedentary behavior were limited to the years in which they were measured. Odds ratios with 95% confidence intervals were adjusted for age, sex, and race or ethnic group. Missing data were not included in the analysis.

All statistical analyses were conducted using R, version 4.1.2 (R Foundation for Statistical Computing, Vienna, Austria), and Joinpoint Regression software, version 4.6.0.0 (Statistical Methodology and Applications Branch, Surveillance Research Program, National Cancer Institute, USA) [13]. Recommended sample weights were used to obtain unbiased estimates. All statistical analyses were two-sided, and significance was defined as *p* < 0.05.

## 3. Results

**Figure 1:**
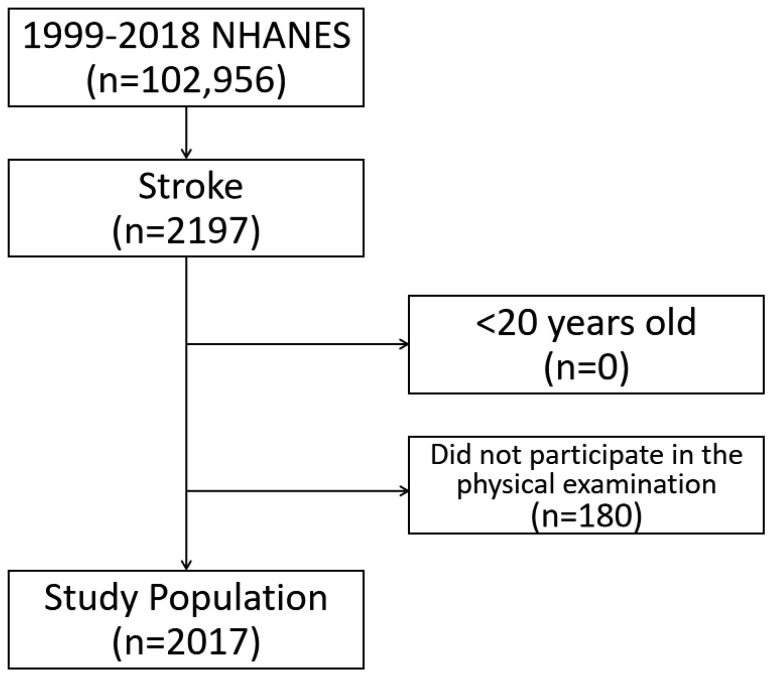
Participant flowchart.

Data for a total of 2017 respondents with a history of stroke were extracted from the 1999 to 2018 NHANESs (Figure 1). The characteristics of the respondents and non-respondents are shown in Supplemental Table S1. Over the course of the surveys, the characteristics of sex, race/ethnicity (except for the other grouping), family income, and marital status remained stable (Table 1). There were positive trends for education and family income, but a reduction in employment.

There were no missing data for age, sex, race/ethnicity, employment status, or smoking. Data were missing for depression in 13.1% of respondents, for alcohol use in 8.8%, for income in 8.1%, for dietary patterns in 8.0%, for obesity in 6.8%, for marital status in 0.8%, for physical activity in 0.6%, for sedentary behavior in 0.8%, and for education in 0.1%. 

### 3.1. Trends in Unhealthy Lifestyle Factors

**Figure 2:**
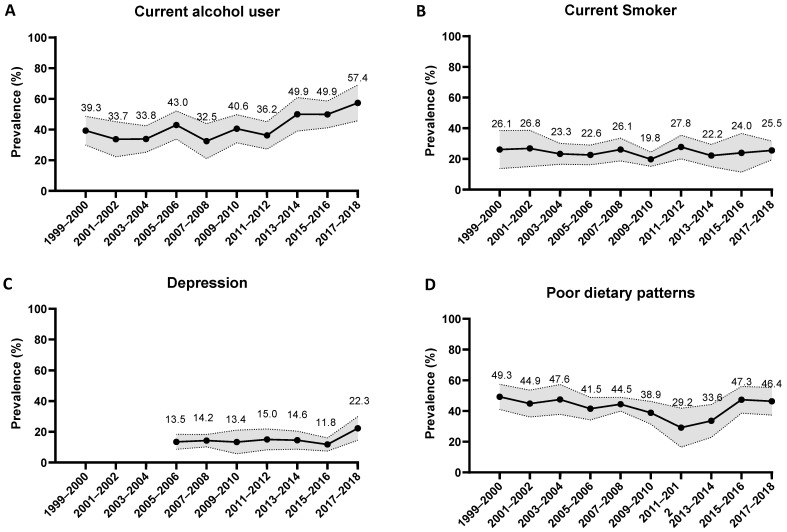

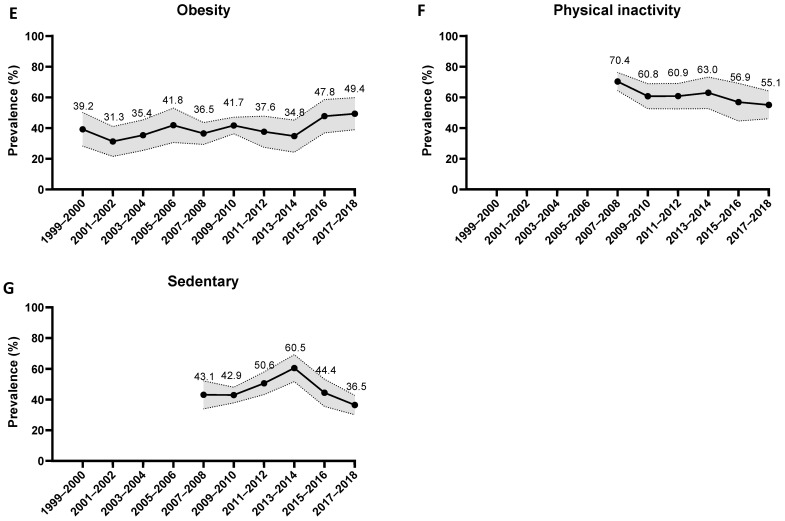
Age-Standardized Trends in Prevalence of Unhealthy Lifestyles among U.S. Adults with a History of Stroke, 1999–2018. Showed as trends for current alcohol drinking (**A**), smoking (**B**), depression (**C**), poor diet (**D**), obesity (**E**), physical inactivity (**F**) and sedentary behavior (**G**). Shaded areas indicate 95% confidence intervals. Representative information for depression was available in the NHANES only from 2005–2006 through 2017–2018. Representative information for the physical inactivity and sedentary factors was available in the NHANES only from 2007–2008 through 2017–2018. NHANES, National Health and Nutrition Examination Survey.

Age-standardized trends in the prevalence of unhealthy lifestyle factors in respondents with a history of stroke are presented in Figure 2 and Supplemental Table S2. Trends for current alcohol drinking, poor diet, and sedentary behavior were nonlinear, with an inflection point around 2011–2014 (Figure 2; Supplemental Tables S2–S4).

Smoking prevalence remained similar across the surveys. The age-adjusted estimated prevalence was 26.1% (95% CI: 13.7 to 38.6) in 1999–2000 and 25.5% (19.4 to 31.6) in 2017–2018, with an AAPC of 0.3% (−3.1 to 3.8; *p* = 0.865). The prevalence of current alcohol drinking significantly increased from 39.3% (29.8 to 48.7) to 57.4% (45.7 to 69.0) over the same period, with an AAPC of 4.9% (1.6 to 8.2; *p* = 0.008). Depression increased non-significantly from 2005–2006 to 2017–2018 (13.5% (8.6 to 18.4) 22.3% (14.6 to 30.0)), with an AAPC of 4.4% (95% −4.7 to 14.4; *p* = 0.277) and with most of the increase occurring from 2015–2016 (11.8% (7.4 to 16.1)). The prevalence of unhealthy diets (AHA Secondary score < 32) declined significantly, from 44.5% (32.4 to 56.5) in 1999–2000 to 29.0% (17.5 to 40.4) in 2011–2012 (APC: −5.9% (−10.1 to −1.5); *p* = 0.019), but then returned to the previous level in 2017–2018 (42.0% (31.4 to 52.7); APC: 9.0% (−7.1 to 28.1); *p* = 0.225). Obesity increased from 39.2% (28.3 to 50.2) in 1999–2000 to 49.4% (38.9 to 59.8) in 2017–2018, with an AAPC of 3.4% (0.4 to 6.4; *p* = 0.029). The prevalence of physical inactivity gradually decreased between 2007–2008 and 2017–2018 (70.4% (64.4 to 76.3) to 55.1% (46.1 to 64.2)), with an AAPC of −4.5% (−7.5 to −1.3; *p* = 0.017). Conversely, the prevalence of sedentary behavior non-significantly increased from 43.1% (34.0 to 52.2) in 2007–2008 to 60.5% (51.7 to 69.3) in 2013–2014 (APC: 14.2% (−46.7 to 144.6); *p* = 0.270) before decreasing to 36.5% (30.1 to 42.8) in 2017–2018 (APC: −21.5% (−84.6 to 301.6)), but with no statistical significance.

**Figure 3:**
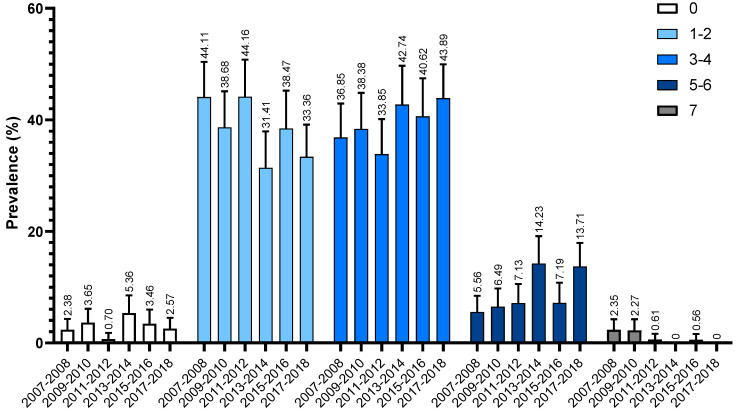
Trends in the Number of Unhealthy Lifestyle Behaviors among U.S. Adults with a History of Stroke, 2007–2018. The total unhealthy lifestyle score for each participant was the number of all unhealthy lifestyle risk factor. The total unhealthy lifestyle score was grouped in 5 groups as “0 = Ideal”, “1–2 = Good”, “3–4= Intermediate”, “5–6 = Poor”, “7 = Extremely poor”.

Changes in the unhealthy lifestyle score over time are shown in Figure 3. The proportion of patients with 7 unhealthy factors (maximum score; considered an extremely poor lifestyle) showed a gradual reduction over time, from 2.3% (0.4 to 4.3) in 2007–2008 to 0 in 2017–2018. The proportion with 5–6 unhealthy factors (poor scores) increased significantly from 5.6% (2.7 to 8.5) in 2007–2008 to 14.2% (9.3 to 19.1) in 2013–2014 (APC: 32.3% (6.1 to 65.1); *p* = 0.04), and then remained at a similar level in 2017–2018 (13.7% (9.5 to 17.9); APC:3.3% (−28.5 to 49.2); *p* = 0.463). The proportion with 3–4 unhealthy factors (intermediate scores) increased non-significantly over time, with an AAPC of 3.7% (−0.8 to 8.5; *p* = 0.085). The proportion with 1–2 unhealthy factors (good) decreased from 2007–2008 to 2017–2018, with an AAPC of −4.9% (−11.2 to 1.9; *p* = 0.116), but with no statistical significance. An “ideal” lifestyle was rare and was found in a similar proportion of respondents in each survey, with an AAPC of 3.2% (−27.2, 46.1; *p* = 0.82).

### 3.2. Risk of Unhealthy Lifestyle by Sociodemographic Factors

The ORs for unhealthy factors by sociodemographic factors are shown in Table 2. Smoking prevalence between 1999–2018 varied significantly by age group, race/ethnicity, family income, education level, and marital status. Respondents aged ≥65 years had a significantly lower risk of smoking than younger respondents (20–44 years). Smoking also showed a step-wise decline with increasing income/education. Respondents who were widowed, divorced, or separated had a significantly higher risk of smoking than those who were married or living with a partner.

The prevalence of current alcohol drinking significantly differed according to age group, sex, family income, education level, and employment status. Respondents who were ≥45 years old or unemployed, were less likely to be current alcohol drinkers than comparator groups. Those who were male, or who had a mid- or upper-range income or education level, were more likely to be alcohol drinkers.

Depression was more common in respondents who were widowed, divorced, or separated than among those who were married or living with partner, as well as in those who were unemployed. Older respondents (≥65 years) or those with a high family income had a lower risk of depression compared to younger or less affluent respondents.

Older respondents (≥65 years), those who were Hispanic, those with a mid- to upper-range family income, or those with a high level of education were less likely to have a unhealthy diet than comparator groups. Non-Hispanic Black respondents and those who where unemployed were more likely to be obese than those of other races/ethnicities or those who were employed.

Physical inactivity and sedentary behavior had significantly different prevalences in respondents according to age, sex, race/ethnicity, family income, education level, and marital status. Both were significantly more common among those aged ≥45 years compared to those in the 20–44-year age group. Additionally, physical inactivity became less common as education level increased. Respondents who were widowed, divorced, separated, or unemployed were less likely to engage in physical exercise compared with those who were married, living with a partner, or employed. Respondents who were ≥45 years old were more likely to have sedentary behavior than those who were 20–44 years old. The risk of sedentary behavior was higher among respondents who were unemployed compared with those who were employed. 

## 4. Discussion

This study examined the prevalence of a number of lifestyle factors among adults with a history of stroke who responded to the U.S. NHANESs between 1999 and 2018, namely smoking, alcohol drinking, depression, unhealthy diet, obesity, physical inactivity, and sedentary behavior. We observed modest reductions in the prevalence of physical inactivity over the surveys, while smoking, alcohol drinking, depression, poor diet, obesity, and sedentary behavior either remained stable or increased. Combined analysis showed that the proportion of patients with three to six of these unhealthy factors seems to have increased over time, demonstrating that these trends did not occur in isolation and suggesting an increasingly unhealthy population. Notably, we found that respondents with a history of stroke and who were widowed, divorced, separated, or unemployed, were at a higher risk of having unhealthy lifestyle factors than those with other martial statuses or who were employed.

The trends in unhealthy lifestyle factors identified in respondents with a history of stroke are roughly in accordance with those noted among the general U.S. population [1]. Physical exercise is one of the most important recommendations for secondary prevention in the current stroke management guidelines [14,15]. Recommendations published in 2004 may have contributed to the reductions in physical inactivity that we observed between 2007 and 2018 [14]. However, with 55% of respondents reporting physical inactivity in the final 2017–2018 survey, there is still much room for improvement. Daily physical activity among stroke survivors has previously been reported to be lower than that in the general population; furthermore, it has also been reported to be lower than in older adults with other chronic health conditions, such as diabetes or cardiovascular disease [16,17]. This suggests that stroke survivors are at particular risk of physical inactivity. However, monitoring of physical activity in patients with a history of stroke is often inadequate, and self-reporting can be inaccurate. Patients who have suffered strokes may be significantly underconditioned and have high activity-related energy costs. Therefore, it is probable that they erroneously believed their levels of exercise were high [18]. 

Although we found physical inactivity to have reduced in respondents with a history of stroke between 2007 and 2014, sedentary behavior seems to have increased. Sedentary behavior can be influenced by a range of factors, including those related to strokes themselves (e.g., stroke severity, comorbid conditions, fear of another stroke, motivation, fatigue, cognition), social/cultural factors (e.g., family support, social policies, professional advice), and environmental factors (e.g., lack of appropriate access to equipment) [19]. It has been reported that active leisure activities decline following strokes, often being replaced with more sedentary activities such as watching television and reading [20]. Reduction in sedentary behavior was included in the 2004 management guidelines, and it was given further emphasis in the 2014 statement by the AHA [14,21]. It is possible that as a result, we observed a trend of a reduction in sedentary behavior in the 2013–2014 survey.

We found trends for stable or increasing prevalence of alcohol drinking, obesity, and poor diet across survey years, paralleling trends in the general U.S. population [1,22]. According to the data estimated from NHANES, the prevalence of obesity among adults increased from 1999 to 2000 through 2013 to 2014, from 30.5% to 37.7%, in the United States [1]. Although stroke patients appear to have higher obesity rates than the general population, both patterns are growing. It is suggested that the poor control of unhealthy lifestyle factors can not only be observed due to the secondary prevention of stroke, but also lacks a gratifying situation in the general population. It was reassuring to see early indications of dietary health among the stroke population in this study. Similar improvement of AHA healthy diet scores was observed in the general U.S. population from 2003 to 2004 and from 2015 to 2016. However, in the population of stroke patients assessed in 2017–2018, we discovered that the prevalence of poor diets had grown once more. This implies that dietary health education has not always been successful among stroke patients, even though data from the general population were inaccessible. The situation of controlling smoking in stroke patients is even more frustrating. Our findings also indicate a stable prevalence of smoking in respondents with a history of stroke over the past 10 years. A similar result was found by another analysis of nationally representative U.S. health survey data from the Centers for Disease Control and Prevention’s Behavioral Risk Factor Surveillance System (BRFSS). The smoking cessation rate among stroke survivors appeared to be worse than among cancer survivors [23]. This is in contrast to trends reported from the general U.S. population, where smoking cessation appears to have increased from 1999–2000 to 2017–2018 [1]. Although national guidelines consistently encourage smoking cessation, there is still a substantial proportion of those with a history of stroke who continue smoking. It is suggested that medical teams and patients themselves make more effort to achieve smoking cessation after a stroke. Publication of national consensus guidelines alone has generally not been sufficient to produce substantial changes in either physician behavior or patient treatment unless both the medical institution and the patient adhere to them [24].

According to our analysis, unemployed respondents with a history of stroke were at an increased risk of multiple unhealthy lifestyle factors, including alcohol drinking, depression, obesity, inadequate physical activity, and sedentary behavior. Many stroke survivors have enduring impairments, such as hemiparesis, spasticity, cognitive dysfunction, and aphasia. More than 30% of stroke survivors report difficulty in fulfilling societal roles for as long as 4 years after a stroke; this can include difficulty retaining employment [25]. Younger stroke survivors are likely to be in an especially socioeconomically demanding phase of life, and have been found to be at a risk of unemployment nine times higher than that of the general population eight years after a stroke [26]. These findings demonstrate the potential long-term employment issues and financial burdens caused by strokes. The limitations of enduring impairments on exercise capacity and the potential for a chronically sedentary lifestyle may be further compounded by the mental challenges of recovery and the notable prevalence of depression among stroke survivors. This must be taken into account when planning stroke rehabilitation and secondary prevention strategies.

Previous studies have shown the positive association between marriage and health outcomes [27]. We found that those who were widowed, divorced, or separated were at a higher risk of several unhealthy lifestyle factors than those who were married. Previous studies have found marital status to be associated with poorer acute ischemic stroke outcomes, including for survival, stroke recurrence, and disability. Being widowed, divorced, or never married was associated with a higher incidence of post-stroke events after 1 year than being married [28]. There may be many reasons that married people often have better health outcomes. Research has shown that married people have better mental health than those who are single, widowed, separated, or divorced [29,30]. It may also be that the presence of a spouse encourages treatment-seeking and adherence to treatment regimes [31], better diet, reduced smoking, and more exercise [32,33]; or provides more stable behavioral and psychosocial influences [34]. However, this has not been found in all studies. It has been reported that unmarried people are at a greater risk of mortality after a stroke than those who have had a marital dissolution [35]. We did not find the same higher risk of unhealthy lifestyle in single people. Williams and Dupre found that continuously unmarried people rated their health similarly to continuously married individuals [36]. Furthermore, although the never married, divorced, and widowed all presumably lack the resources that marriage provides, it is only the previously married who appear to be psychologically disadvantaged by being unmarried [37], suggesting that marital factors may possess further complex effects on healthy lifestyle control after the onset of a major illness.

There remains a lack of strong evidence showing improvements in stroke outcomes with current comprehensive interventions [38]. In terms of primary stroke prevention, one epidemiological study reported that a combination of four health behaviors (i.e., physical activity, current non-smoking, moderate alcohol intake, and adequate daily vitamin C intake) was associated with a two-fold reduction in the incidence of stroke [39]. 

Lifestyle likely influences the risk of stroke in part through clinical risk factors, including atherosclerosis, hypertension, and diabetes [40,41]. Healthy behaviors are often interlinked in their occurrence, and increased emphasis on health awareness is of high importance in the secondary prevention of vascular events after stroke [42]. A multifaceted approach to stroke management, including non-pharmacological interventions (i.e., exercise, dietary advice, lifestyle counseling, and patient education) and appropriate pharmacological therapy, has been encouraged for many years [5,43]. Despite this, we have found that the proportion of people with a history of stroke in the U.S. with unhealthy lifestyle factors is increasing. Developing effective public health policy depends upon intersectoral action. Full weight must be given to health considerations in policy-making across different sectors that affect health (education, public safety, housing, etc.) A better understanding of these factors may aid improvements in public health policy. Our findings may also help better understand the sociodemographic barriers to a healthy lifestyle.

### Limitations

Our study has some limitations. Firstly, because of the relatively small sample, we may have lacked the statistical power to detect small changes in lifestyle control among respondents with a history of stroke. Secondly, NHANES data are largely self-reported and are subject to random and systematic reporting errors. Thirdly, smoking was not validated with biochemical tests, and self-reporting may understate the actual prevalence. Similarly, self-reported dietary information is subject to measurement error and misclassification. NHANES incorporates 2 standardized 24 h diet recalls per person, which were energy-adjusted and averaged whenever possible to reduce measurement error. Fourth, the sedentary lifestyle could be a sequelae after stroke. Because the NHANES study did not provide a specific stroke burden to stratify participants, the results of the sedentary lifestyle analysis may have been exaggerated. Fifth, the diagnosis and treatment of stroke have advanced with the advancement of imaging and the increased focus on the public’s ability to recognize strokes, which may change the sequelae and illness severity of the stroke population in recent years from what it was previously. This may be a confounder when analyzing trends. For instance, more stroke patients may have received treatment more quickly and with fewer sequelae in 2018 than in 1999, hence improving their physical inactivity. Meanwhile, some clinical complications such as atrial fibrillation, hypertension, or diabetes are also risk factors associated with stroke, and these may be the confounder factors. Further analysis can be performed in the future according to different stratifications of disease severity, and to understand the impact of these confounding factors on trends.

## 5. Conclusions

Using data from NHANESs from 1999 to 2018, we found an overall increase in unhealthy lifestyle factors among respondents with a history of stroke. In particular, while the prevalence of smoking and depression generally remained stable, alcohol drinking increased significantly. Poor diet initially decreased before returning to its initial prevalence. Physical inactivity gradually decreased, while sedentary behavior marginally increased before decreasing. The proportion of respondents with 3–6 unhealthy lifestyle factors increased over time. Respondents who were widowed, divorced, separated, or unemployed were at a higher risk of having multiple unhealthy lifestyle factors.

## Figures and Tables

**Table 1:** Characteristics of Adults with a History of Stroke in the NHANES, 1999 to 2018.

Characteristics *	1999–2000	2001–2002	2003–2004	2005–2006	2007–2008	2009–2010	2011–2012	2013–2014	2015–2016	2017–2018
	*n* = 157	*n* = 159	*n* = 194	*n* = 185	*n* = 241	*n* = 218	*n* = 216	*n* = 194	*n* = 198	*n* = 255
**Age % (95% CI)**										
20–44 years	12.8 (4.2–21.4)	17.8 (8.3–27.4)	15.8 (7.6–24.1)	8.5 (3.2–13.8)	9.3 (3.4–15.3)	10.1 (4.3–16.0)	9.4 (3.7–15.2)	6.6 (2.9–10.3)	12.2 (5.5–18.9)	5.2 (1.9–8.6)
45–64 years	30.0 (18.6–41.3)	30.8 (18.0–43.5)	30.7 (23.6–37.7)	33.5(24.2–42.7)	33.1 (24.0–42.1)	35.3 (27.5–43.1)	35.5 (29.2–41.8)	31.8 (23.9–39.7)	33.1 (24.6–41.7)	40.6 (31.2–50.0)
≥65 years	57.2 (47.2–67.2)	51.4 (40.4–62.4)	53.5 (44.6–62.4)	58.0 (47.4–68.7)	57.6 (49.2–66.0)	54.6 (44.0–65.2)	55.1 (50.4–59.7)	61.6 (53.9–69.3)	54.7 (44.9–64.5)	54.1 (43.7–64.6)
**Sex % (95% CI)**										
Male	44.1 (35.2–52.9)	44.8 (33.4–56.2)	39.5 (33.4–45.6)	39.7 (30.6–48.8)	41.4 (32.3–50.4)	46.2 (37.8–54.5)	44.5 (36.0–53.0)	40.6 (29.8–51.5)	51.5 (44.0–59.0)	41.2 (33.1–49.4)
Female	55.9 (47.1–64.8)	55.2 (43.8–66.6)	60.5 (54.4–66.6)	60.3 (51.2–69.4)	58.6 (49.6–67.7)	53.8 (45.5–62.2)	55.5 (47.0–64.0)	59.4 (48.5–70.2)	48.5 (41.0–56.0)	58.8 (50.6–66.9)
**Race/ethnicity % (95% CI) †**										
Non-Hispanic White	73.2 (64.0–82.4)	71.0 (60.9–81.1)	73.1 (64.2–82.1)	75.4 (65.5–85.3)	74.5 (64.2–84.8)	70.0 (62.0–78.0)	62.6 (53.5–71.6)	74.6 (68.7–80.6)	61.7 (47.2–76.3)	64.4 (54.1–74.7)
Non-Hispanic Black	13.1 (7.6–18.6)	16.5 (8.8–24.2)	14.3 (8.0–20.6)	13.6 (7.9–19.3)	16.0 (9.9–22.1)	16.6 (10.6–22.7)	17.9 (8.3–27.5)	11.4 (7.3–15.5)	16.0 (8.4–23.6)	18.3 (12.3–24.3)
Hispanic	10.2 (3.3–17.1)	8.3 (−1.4–18.1)	4.6 (−0.1–9.3)	6.2 (1.6–10.8)	5.2 (1.6–8.7)	7.1 (1.5–12.7)	10.3 (5.7–14.9)	7.5 (4.3–10.8)	10.3 (6.3–14.2)	7.4 (3.9–10.8)
Other (Non-Hispanic Asian, Mexican-American, multi-racial, or other race) ‡	3.5 (−0.3–7.3)	4.2 (−0.6–8.9)	8.0 (2.2–13.8)	4.8 (0.5–9.1)	4.3 (0.0–8.6)	6.3 (3.0–9.5)	9.2 (5.0–13.4)	6.4 (2.4–10.4)	12.0 (3.3–20.6)	9.9 (4.5–15.3)
**Ratio of family income to poverty level % (95% CI)**										
<1.30	33.7 (22.6–44.8)	32.8 (17.5–48.1)	31.3 (24.5–38.0)	25.4 (15.1–35.6)	27.2 (18.3–36.0)	27.0 (19.0–35.0)	36.2 (29.6–42.9)	30.4 (19.7–41.1)	33.4 (23.5–43.3)	26.7 (21.1–32.3)
1.30–3.49	31.0 (22.8–39.2)	32.8 (21.8–43.8)	42.9 (35.3–50.5)	47.5 (39.4–55.6)	43.8 (31.5–56.0)	39.5 (31.2–47.8)	38.1 (30.7–45.4)	44.6 (34.4–54.8)	37.2 (29.2–45.3)	37.0 (28.1–45.9)
≥3.50	23.9 (16.1–31.7)	27.1 (18.9–35.3)	19.9 (12.7–27.1)	20.3 (14.0–26.6)	19.0 (12.9–25.0)	27.6 (17.8–37.4)	21.8 (14.1–29.5)	17.0 (10.9–23.1)	22.2 (11.7–32.7)	24.2 (15.4–33.1)
**Education level % (95% CI)**										
Below high school	32.7 (24.4–40.9)	40.6 (30.7–50.5)	36.8 (28.1–45.5)	27.0 (20.0–34.1)	32.5 (26.1–39.0)	30.5 (22.5–38.5)	31.3 (23.5–39.2)	28.3 (20.9–35.7)	20.1 (13.3–26.9)	17.2 (10.4–24.0)
High school graduate or GED	35.1 (26.1–44.2)	22.6 (15.2–30.1)	24.8 (19.4–30.2)	25.8 (17.1–34.4)	32.3 (25.4–39.2)	26.1 (17.7–34.5)	28.7 (21.2–36.3)	23.7 (15.0–32.4)	33.8 (26.5–41.1)	39.9 (33.2–46.7)
Some college or above	32.2 (23.4–41.1)	36.7 (27.1–46.4)	37.6 (29.3–46.0)	47.2 (36.7–57.7)	35.2 (26.6–43.7)	43.4 (30.4–56.5)	39.7 (31.6–47.8)	47.9 (37.8–58.0)	46.1 (36.6–55.6)	42.9 (33.1–52.6)
**Marital status % (95% CI)**										
Married or living with partner	48.7 (36.8–60.5)	60.1 (51.6–68.6)	53.2 (43.1–63.3)	55.1 (46.0–64.2)	55.7 (49.2–62.2)	59.5 (50.5–68.4)	51.9 (41.6–62.2)	54.0 (44.2–63.8)	53.1 (43.0–63.1)	57.7 (46.0–69.4)
Never married	4.9 (−0.1–9.9)	8.6 (3.1–14.2)	9.6 (3.7–15.4)	4.0 (1.6–6.4)	8.8 (3.1–14.4)	8.5 (3.2–13.9)	7.4 (3.0–11.8)	9.2 (5.7–12.7)	12.6 (7.5–17.8)	5.5 (3.2–7.8)
Widowed, divorced, or separated	36.2 (26.6–45.7)	31.3 (23.0–39.6)	36.9 (27.4–46.4)	40.9 (31.5–50.2)	35.6 (27.1–44.1)	32.0 (26.0–38.0)	40.7 (32.6–48.8)	36.8 (27.9–45.8)	34.3 (25.6–43.0)	36.5 (25.5–47.4)
**Employment status % (95% CI)**										
Employed	22.4 (10.2–34.6)	23.9 (11.7–36.2)	23.4 (16.0–30.8)	18.8 (11.1–26.5)	22.5 (15.1–29.9)	21.6 (13.7–29.5)	19.2 (11.9–26.6)	14.9 (9.3–20.4)	16.5 (8.8–24.1)	18.3 (10.7–25.9)
Unemployed	77.6 (65.4–89.8)	76.1 (63.8–88.3)	76.6 (69.2–84.0)	81.2 (73.5–88.9)	77.5 (70.1–84.9)	78.4 (70.5–86.3)	80.8 (73.4–88.1)	85.1 (79.6–90.7)	83.5 (75.9–91.2)	81.7 (74.1–89.3)

* The sample size for each 2-year interval is unweighted, but all other numbers in the table are weighted percentages (with 95% confidence intervals). † Race or ethnic group was reported by the participants. ‡ Representative information for non-Hispanic Asian Americans was available in the NHANES only from 2011 through 2018. CI, confidence interval; GED, General Equivalent Diploma; NHANES, National Health and Nutrition Examination Survey.

**Table 2:** Adjusted Odds Ratios for Unhealthy Lifestyle Behaviors/Characteristics among U.S. Adults with a History of Stroke, 1999–2018.

Variable †	Current Smoker	Current Alcohol Drinker	Depression ‡	Unhealthy Diet	Obesity	Physical Inactivity ‡	Sedentary Behavior
**Age**							
20–44	1 (reference)	1 (reference)	1 (reference)	1 (reference)	1 (reference)	1 (reference)	1 (reference)
45–64	0.89 (0.54–1.46)	0.52 (0.32–0.83) *	0.99 (0.50–1.93)	0.76 (0.44–1.30)	1.29 (0.83–2.00)	2.03 (1.09–3.76) *	1.85 (1.11–3.10) *
≥65	0.16 (0.10–0.26) *	0.27 (0.17–0.42) *	0.30 (0.16–0.55) *	0.41 (0.24–0.68) *	0.73 (0.48–1.10)	4.13 (2.54–6.73) *	2.23 (1.27–3.91) *
**Sex**							
Female	1 (reference)	1 (reference)	1 (reference)	1 (reference)	1 (reference)	1 (reference)	1 (reference)
Male	1.06 (0.80–1.39)	1.83 (1.41–2.37) *	0.77 (0.51–1.16)	1.36 (1.02–1.79) *	1.03 (0.82–1.30)	0.68 (0.51–0.89) *	1.14 (0.86–1.52)
**Race/ethnicity**							
Non-Hispanic White	1 (reference)	1 (reference)	1 (reference)	1 (reference)	1 (reference)	1 (reference)	1 (reference)
Non-Hispanic Black	1.01(0.75–1.38)	0.77(0.57–1.03)	0.84(0.58–1.23)	0.93(0.72–1.18)	1.40(1.05–1.85) *	1.16(0.86–1.56)	0.85(0.63–1.16)
Hispanic	0.53(0.31–0.89) *	0.74(0.54–1.03)	0.90(0.57–1.44)	0.67(0.48–0.95) *	1.21(0.85–1.73)	1.34(0.92–1.95)	0.46(0.30–0.68) *
Other	1.33(0.66–2.68)	0.79(0.45–1.37)	0.80(0.36–1.77)	0.68(0.41–1.12)	0.88(0.54–1.42)	0.66(0.36–1.20)	0.95(0.59–1.53)
**Ratio of family income to poverty level (PIR) %**							
<1.30	1 (reference)	1 (reference)	1 (reference)	1 (reference)	1 (reference)	1 (reference)	1 (reference)
1.30–3.49	0.53 (0.38–0.73) *	1.55 (1.13–2.13) *	0.98 (0.63–1.53)	0.67 (0.49–0.92) *	1.25 (0.93–1.68)	0.75 (0.51–1.09)	0.93 (0.64–1.36)
≥3.50	0.28 (0.17–0.46) *	2.88 (1.94–4.28) *	0.40 (0.20–0.81) *	0.57 (0.40–0.81) *	1.03 (0.70–1.52)	0.45 (0.29–0.69) *	1.42 (0.92–2.19)
**Education level**							
Below high school	1 (reference)	1 (reference)	1 (reference)	1 (reference)	1 (reference)	1 (reference)	1 (reference)
High school graduate or GED	0.60 (0.42–0.86) *	1.71 (1.24–2.37) *	0.94 (0.62–1.42)	0.81 (0.60–1.09)	1.32 (0.97–1.80)	0.62 (0.42–0.93) *	0.87 (0.60–1.26)
Some college or above	0.36 (0.26–0.51) *	2.49 (1.84–3.37) *	0.66 (0.42–1.04)	0.55 (0.40–0.76) *	0.99 (0.72–1.35)	0.45 (0.32–0.63) *	0.99 (0.68–1.44)
**Marital status**							
Married or living with a partner	1 (reference)	1 (reference)	1 (reference)	1 (reference)	1 (reference)	1 (reference)	1 (reference)
Never married	1.17 (0.68–1.99)	0.98 (0.58–1.65)	1.58 (0.93–2.69)	0.94 (0.55–1.62)	0.74 (0.47–1.15)	1.20 (0.72–2.01)	0.87 (0.49–1.54)
Widowed, divorced, or separated	1.52 (1.10–2.10) *	1.04 (0.80–1.36)	1.54 (1.01–2.35) *	1.27 (0.95–1.69)	0.84 (0.64–1.08)	1.42 (1.03–1.96) *	1.25 (0.92–1.69)
**Employment status**							
Employed	1 (reference)	1 (reference)	1 (reference)	1 (reference)	1 (reference)	1 (reference)	1 (reference)
Unemployed	1.17 (0.78–1.77)	0.64 (0.46–0.91) *	2.79 (1.49–5.22) *	0.96 (0.67–1.37)	1.46 (1.01–2.09) *	2.96 (1.83–4.79) *	1.64 (1.03–2.61) *

† Odds ratio with 95% confidence intervals were adjusted for age, sex, and race or ethnic group. ‡ Data for depression are only available from 2005 through 2018; data for physical inactivity and sedentary are only available from 2007 through 2018. * *p* < 0.05. GED denotes General Equivalent Diploma; PIR, poverty to income ratio.

## Data Availability

Data available on request due to restrictions e.g., privacy or ethical.

## Supplementary Materials

The following supporting information can be downloaded at: https://www.mdpi.com/article/10.3390/jcm12031223/s1, Table S1: Characteristics of respondents and no-respondents; Table S2: Age-standardized prevalence of unhealthy lifestyle behaviors among U.S. adults with a history of stroke, 1999–2018; Table S3: Mean PHQ-9 score, AHA score, BMI, physical activity and sitting time in adults with a history of stroke, 1999–2018; Table S4: Annual rate of change before and after inflection point in unhealthy lifestyles among adults with a history of stroke, 1999–2018.
