# Supplementary material for: Trends in Unhealthy Lifestyle Factors among Adults with Stroke in the United States between 1999 and 2018
Source: J Clin Med. 2023 Feb 3;12(3):1223. doi: 10.3390/jcm12031223 (PMC9917618; doi:10.3390/jcm12031223)
Supplement: Supplementary file 1 [file jcm-12-01223-s001.zip › jcm-2195263-supplementary.pdf]

Supplemental Table S1. Characteristics of respondents and no-respondents

Characteristics	Respondents	No-respondents	*p*
	n=2,017	n=180	
**Age % (95% CI)**			<0.001
20-44 yr	5.9(4.9-7)	1.1(-0.4-2.6)	
45-64 yr	30.8(28.8-32.9)	14.4(9.3-19.6)	
⩾65 yr	63.2(61.1-65.3)	84.4(79.1-89.7)	
**Sex % (95% CI)**			0.47
Male	49.5(47.3-51.7)	46.7(39.4-54)	
Female	50.5(48.3-52.7)	53.3(46-60.6)	
**Race/ethnicity % (95% CI)†**			0.046
Non-Hispanic White	50.5(48.3-52.7)	60.6(53.4-67.7)	
Non-Hispanic Black	26.8(24.9-28.8)	18.3(12.7-24)	
Hispanic	5.1(4.1-6)	4.4(1.4-7.5)	
Other (Non-Hispanic Asian ,Mexican-American, multi- racial, and other race)‡	17.6(15.9-19.3)	16.7(11.2-22.1)	
**Ratio of family income to poverty level % (95% CI)**			0.077
＜1.30	36.6(34.5-38.7)	28.9(22.3-35.5)	

1.30-3.49	46.8(44.6-49)	50(42.7-57.3)	
⩾3.50	16.6(14.9-18.2)	21.1(15.1-27.1)	
**Education level % (95% CI)**			0.136
Below High School	38.3(36.2-40.4)	45(37.7-52.3)	
High School graduate or GED	25.7(23.8-27.6)	22.2(16.1-28.3)	
Some college or above 45	35.8(33.7-37.9)	30.6(23.8-37.3)	
**Marital status % (95% CI)**			0.011
Married or living with partner 16	50.3(48.1-52.5)	40.6(33.4-47.7)	
Never married 5	7.8(6.7-9)	5.6(2.2-8.9)	
Widowed, divorced, or separated 234	40.9(38.7-43)	51.7(44.4-59)	
**Employment status % (95% CI)**			0.016
Employed	14.3(12.8-15.9)	7.8(3.9-11.7)	
Unemployed	85.7(84.1-87.2)	91.7(87.6-95.7)	

† Race or ethnic group were reported by the participants.

‡ Representative information for non-Hispanic Asian Americans was available in the NHANES only from 2011 through 2018.

CI, confidence interval; GED, General Equivalent Diploma.

Supplemental Table S2. Age-Standardized Prevalence of Unhealthy Lifestyle Behaviors Among U.S. Adults with a History of Stroke, 1999–2018

	Adults with stroke, % (95% CI)									
	1999-2000	2001-2002	2003-2004	2005-2006	2007-2008	2009-2010	2011-2012	2013-2014	2015-2016	2017-2018
Current smoking	26.1 (13.7-38.6)	26.8 (15.1-38.6)	23.3 (16.5-30.1)	22.6 (16.2-29.1)	26.1 (18.7-33.5)	19.8 (15.2-24.5)	27.8 (20.1-35.4)	22.2 (15.0-29.4)	24.0 (11.3-36.6)	25.5 (19.4-31.6)
Current alcohol drinker	39.3 (29.8-48.7)	33.7 (22.3-45.1)	33.8 (25.2-42.5)	43.0 (33.8-52.1)	32.5 (21.1-43.9)	40.6 (31.4-49.7)	36.2 (27.3-45.1)	49.9 (39.0-60.9)	49.9 (41.1-58.6)	57.4 (45.7-69.0)
Depression*	-	-	-	13.5 (8.6-18.4)	14.3 (10.2-18.3)	13.4 (5.7-21.1)	15.0 (8.3-21.8)	14.6 (8.7-20.4)	11.8 (7.4-16.1)	22.3 (14.6-30.0)
Unhealthy diet	44.5 (32.4-56.5)	42.0 (32.8-51.3)	39.7 (31.1-48.2)	40.8 (30.7-50.9)	39.2 (30.5-47.8)	31.1 (23.4-38.9)	29.0 (17.5-40.4)	36.1 (24.1-48.1)	33.9 (21.1-46.7)	42.0 (31.4-52.7)
Obesity	39.2 (28.3-50.2)	31.3 (21.6-41.0)	35.4 (25.4-45.4)	41.8 (30.7-53.0)	36.5 (29.4-43.6)	41.7 (36.3-47.1)	37.6 (27.5-47.8)	34.8 (24.3-45.2)	47.8 (36.9-58.6)	49.4 (38.9-59.8)
Physical inactivity†	-	-	-	-	70.4 (64.4-76.3)	60.8 (52.6-69.0)	60.9 (52.6-69.1)	63.0 (52.7-73.3)	56.9 (44.6-69.2)	55.1 (46.1-64.2)
Sedentary behavior†	-	-	-	-	43.1 (34.0-52.2)	42.9 (37.9-48.0)	50.6 (43.3-58.0)	60.5 (51.7-69.3)	44.4 (35.6-53.3)	36.5 (30.1-42.8)

CI, confidence interval.

*Information for depression was available from 2005-2006 through 2017-2018.

†Information for physical inactivity and sedentary behavior was available from 2007-2008 through 2017-2018.

Supplemental Table S3: Mean PHQ-9 Score, AHA Score, BMI, Physical Activity and Sitting Time in Adults with a History of Stroke, 1999-2018.

	Adults with stroke, mean (95% CI)									
	1999-2000	2001-2002	2003-2004	2005-2006	2007-2008	2009-2010	2011-2012	2013-2014	2015-2016	2017-2018
PHQ-9 score*	-	-	-	4.5 (3.7-5.4)	5.3 (4.6-6.0)	4.8 (3.5-6.1)	4.5 (3.2-5.8)	4.8 (3.9-5.8)	4.8 (4.2-5.3)	5.8 (4.7-6.9)
AHA diet score	19.9 (18.5-21.2)	19.1 (17.1-21.0)	20.4 (18.5-22.3)	20.4 (18.6-22.2)	20.7 (19.5-21.9)	22.7 (21.4-23.9)	20.8 (19.2-22.5)	21.6 (20.3-22.9)	19.8 (17.2-22.4)	21.9 (20.6-23.3)
BMI	29.7 (28.3-31.0)	29.4 (28.3-30.5)	29.0 (27.8-30.3)	30.2 (29.0-31.4)	29.3 (28.5-30.0)	30.5 (29.6-31.3)	28.6 (27.0-30.2)	29.2 (27.6-30.8)	30.8 (29.3-32.3)	30.9 (29.8-32.1)
Physical activity time†	-	-	-	-	343.6 (194.2-492.9)	435.2 (252.8-617.7)	409.8 (204.6-614.9)	424.1 (285.1-563.1)	593.9 (250.5-937.3)	502.3 (381.4-623.3)
Sitting time†	-	-	-	-	6.4 (5.9-6.9)	6.6 (6.1-7.0)	7.2 (6.5-7.9)	7.8 (7.1-8.4)	7.2 (6.4-7.9)	6.4 (5.8-7.1)

*PHQ-9 score for depression was available from 2005-2006 through 2017-2018.

†Physical activity and sitting times were available from 2007-2008 through 2017-2018. Physical activity times was expressed by equivalent physical activity times (moderate activity time+2×vigorous activity time).

AHA, American Heart Association; BMI, body-mass index; CI, confidence interval; NHANES, National Health and Nutrition Examination Survey; PHQ, Patient Health Questionnaire.

Supplemental Table S4: Annual Rate of Change Before and After Inflection Point in Unhealthy Lifestyles Among Adults with a History of Stroke, 1999–2018

	Inflection point	Annual rate of change before inflection, %/year (95% CI)	Annual rate of change after inflection, %/year (95% CI)	Average annual percentage change
Current Smoker	-	-	-	0.3 (-3.1,3.8)
Current alcohol drinker	-	-	-	4.9 (1.6-8.2)*
Depression	-	-	-	4.4 (-4.7,14.4)
Unhealthy diet	2011-2012	-5.9 (-10.1,-1.5)*	9.0 (-7.1,28.1)	-1.2 (-5.7,3.6)
Obesity	-	-	-	3.4 (0.4,6.4)*
Physical inactivity†	-	-	-	-4.5 (-7.5,-1.3)*
Sedentary behavior†	2013-2014	14.2 (-46.7,144.6)	-21.5 (-84.6,301.6)	-1.7 (-13.0,11.2)

CI, confidence interval

* p<0.05
